# Predicting the tissue outcome of acute ischemic stroke from acute 4D computed tomography perfusion imaging using temporal features and deep learning

**DOI:** 10.3389/fnins.2022.1009654

**Published:** 2022-11-04

**Authors:** Anthony J. Winder, Matthias Wilms, Kimberly Amador, Fabian Flottmann, Jens Fiehler, Nils D. Forkert

**Affiliations:** ^1^Department of Radiology, University of Calgary, Calgary, AB, Canada; ^2^Hotchkiss Brain Institute, University of Calgary, Calgary, AB, Canada; ^3^Department of Paediatrics, Cumming School of Medicine, University of Calgary, Calgary, AB, Canada; ^4^Department of Community Health Sciences, Cumming School of Medicine, University of Calgary, Calgary, AB, Canada; ^5^Alberta Children’s Hospital Research Institute, University of Calgary, Calgary, AB, Canada; ^6^Department of Diagnostic and Interventional Neuroradiology, University Medical Center Hamburg-Eppendorf, Hamburg, Germany; ^7^Department of Clinical Neurosciences, University of Calgary, Calgary, AB, Canada

**Keywords:** stroke, ischemic stroke, brain ischemia, deep learning, precision medicine, perfusion CT, prediction

## Abstract

Predicting follow-up lesions from baseline CT perfusion (CTP) datasets in acute ischemic stroke patients is important for clinical decision making. Deep convolutional networks (DCNs) are assumed to be the current state-of-the-art for this task. However, many DCN classifiers have not been validated against the methods currently used in research (random decision forests, RDF) and clinical routine (Tmax thresholding). Specialized DCNs have even been designed to extract complex temporal features directly from spatiotemporal CTP data instead of using standard perfusion parameter maps. However, the benefits of applying deep learning to source or deconvolved CTP data compared to perfusion parameter maps have not been formally investigated so far. In this work, a modular UNet-based DCN is proposed that separates temporal feature extraction from tissue outcome prediction, allowing for both model validation using perfusion parameter maps as well as end-to-end learning from spatiotemporal CTP data. 145 retrospective datasets comprising baseline CTP imaging, perfusion parameter maps, and follow-up non-contrast CT with manual lesion segmentations were assembled from acute ischemic stroke patients treated with intravenous thrombolysis alone (IV; *n* = 43) or intra-arterial mechanical thrombectomy (IA; *n* = 102) with or without combined IV. Using the perfusion parameter maps as input, the proposed DCN (mean Dice: 0.287) outperformed the RDF (0.262) and simple Tmax-thresholding (0.249). The performance of the proposed DCN was approximately equal using features optimized from the deconvolved residual curves (0.286) compared to perfusion parameter maps (0.287), while using features optimized from the source concentration-time curves (0.296) provided the best tissue outcome predictions.

## Introduction

Although stroke remains the third leading cause of death and disability worldwide, its global burden of disease has decreased dramatically over the last several years owing to medical advancements in acute stroke management (Feigin et al., 2021). In the context of acute ischemic stroke (AIS), which accounts for approximately 90% of stroke cases among high-income countries (O’ Donnell et al., 2010; Virani et al., 2020), these advancements have primarily included: (1) the validation and introduction of novel therapeutic devices, such as next-generation stent retrievers (Goyal et al., 2016), and (2) the refinement of clinical decision rules to expand the use of these novel devices to new patient populations (Albers et al., 2018; Desai et al., 2018). With machine learning becoming increasingly ubiquitous in medical research (Lo Vercio et al., 2020; MacEachern and Forkert, 2021), recent studies concerning both aspects of AIS management have explored the benefits of using supervised classification methods to model the evolution of the ischemic lesion (Yedavalli et al., 2021).

Our current understanding of the progression of ischemic stroke is largely informed by computed tomography perfusion (CTP) and perfusion-weighted magnetic resonance imaging (PWI). Both modalities use time-resolved imaging in conjunction with an intravascular contrast agent to visualize the blood flow through the brain. The temporal signal, or concentration-time curve, measured in a voxel containing ischemic tissue will typically show a delayed and prolonged enhancement, giving some indication of the initial location and severity of ischemia (Demeestere et al., 2020). For ease of use, concentration-time curves are normally not interpreted directly, but rather processed to extract standard temporal features known as perfusion parameters that represent key aspects of the patient’s cerebral hemodynamics (Demeestere et al., 2020). Predetermined thresholds are then applied to patients’ perfusion (and diffusion in case of MRI) parameter maps to differentiate the irreversibly damaged “core” tissue from the potentially salvageable “penumbral” tissue. The typical progression of AIS is for the infarct core to expand into the ischemic penumbra over time, with the final infarct volume depending jointly on the duration, treatment success, and severity of the stroke (Demeestere et al., 2020). Accordingly, the primary aim of acute stroke therapy is to reperfuse the penumbral tissue as immediately and completely as possible.

Large-scale clinical trials (Lansberg et al., 2012; Goyal et al., 2016; Desai et al., 2018) and medical practitioners (Powers et al., 2018) use patients’ core and penumbral tissue volumes to determine whether the potential tissue salvage associated with the use of a therapeutic device justifies the cost and potential risks of treatment. For example, among patients who cannot be treated within 6 h from the onset of AIS, a small infarct core and comparatively large ischemic penumbra resulting from a large vessel occlusion predicts that treatment with a stent retriever device or aspiration catheter, referred to as intra-arterial mechanical thrombectomy (IA), will lead to improved clinical outcomes compared to treatment with intravenous tPA (IV) alone (Powers et al., 2018). Despite the widespread use of threshold-based core and penumbral tissue volumes for treatment decision making, this approach has a number of noteworthy and well-described limitations (Goyal et al., 2020). Applying the same set of thresholds across the whole brain of all patients fails to account for patient-specific (Eilaghi et al., 2014; d’Esterre et al., 2015; Ernst et al., 2015; Bahouth et al., 2018; Winder et al., 2019) and tissue-specific (Payabvash et al., 2011; Chen et al., 2019) factors that affect the tissue’s vulnerability to ischemia. Furthermore, it is difficult to portray the difference in tissue salvage between several possible therapies using a single core and penumbral segmentation.

An alternative approach is to directly predict the patient’s voxel-wise tissue fate (i.e., infarct vs. non-infarct) given a specific set of treatment conditions using machine learning models, which are inherently multivariate and well-suited to large datasets. This paradigm is commonly referred to as “tissue outcome prediction.” To date, several classical machine learning architectures have been evaluated for stroke tissue outcome prediction, including generalized linear models (Kidwell et al., 2013; Kemmling et al., 2015; Flottmann et al., 2017), nearest-neighbor approaches (Gottrup et al., 2005), and random decision forests (McKinley et al., 2017). Generally, comparative studies show decision forests to lead to the best results among classical machine learning models (McKinley et al., 2017; Winder et al., 2019).

In the context of validating novel therapeutic devices, it has been proposed that tissue outcome predictions could be used as a virtual endpoint for *in silico* clinical trials (Winder et al., 2021). The ERASER clinical trial, for example, validated a novel stent retrieval device using random forests to show a reduced predicted infarct volume for IA treatment compared to the true IV treatment outcome (Fiehler et al., 2019). Random decision forests were also used to confirm the non-efficacy of theophylline as a neuroprotective add-on to IV treatment following mixed results in randomized clinical trials (Modrau et al., 2021). Because *in silico* studies such as ERASER have been reported to require far fewer prospectively collected datasets, there is the potential for these tissue outcome prediction models to accelerate the pace and reduce the cost of developing novel devices and treatment approaches for AIS patients.

In the context of refining the patient selection criteria for advanced stroke therapies, such as IA, it has been proposed that machine learning models could improve patient outcomes by helping clinicians to make highly individualized treatment recommendations. To date, the primary focus of tissue outcome prediction studies has been to establish a “state-of-the-art” machine learning model considering both the available classical models (Gottrup et al., 2005; Kidwell et al., 2013; Kemmling et al., 2015; Flottmann et al., 2017; McKinley et al., 2017) and more novel “deep learning” models (Nielsen et al., 2018; Winzeck et al., 2018; Ho et al., 2019; Robben et al., 2020; Yu et al., 2020; Amador et al., 2022). One major hurdle in establishing a state-of-the-art deep learning method has been that machine learning methods are highly data-dependent. As a result, common performance metrics such as Dice and volume error are not usually directly comparable between studies. The ischemic lesion segmentation (ISLES) 2017 challenge (Winzeck et al., 2018) attempted to address this issue by comparing multiple tissue outcome prediction models using the same CTP image data. However, all of the models submitted to the challenge used deep learning architectures, making it impossible to determine whether the top-performing models truly achieve state-of-the-art performance compared to leading classical machine learning models. Moreover, the number of datasets provided by the challenge was still rather small for deep learning applications. Most recent deep learning studies have primarily used simple linear models (Robben et al., 2020; Yu et al., 2020) and single-parameter thresholding (Yu et al., 2020) for comparison, both of which are routinely outperformed by random decision forests (McKinley et al., 2017; Benzakoun et al., 2021). Therefore, the potential for deep learning methods to improve the management of AIS remains to be properly evaluated using appropriate comparison methods.

Compared to classical machine learning, deep learning is unique in that it does not only perform tissue outcome prediction, but can also optimize the feature extraction needed for this task. Since the calculation of perfusion parameter maps can be seen as a form of handcrafted feature extraction, a few studies have proposed to apply deep learning models directly to the concentration-time curves of patients’ MR perfusion (Ho et al., 2019) or CT perfusion (Robben et al., 2020; Amador et al., 2022) datasets instead of the typical perfusion parameter maps. Although the classical perfusion parameters (CBF, CBV, MTT, Tmax) represent meaningful hemodynamic properties to clinicians, whether or not they are the ideal features for tissue outcome prediction and contain all valuable information captured in time-resolved perfusion scans has not been well-studied to date. From this perspective, allowing deep learning models to optimize their own temporal features in a fully data-driven manner may enable them to make better tissue outcome predictions. Furthermore, learning directly from source perfusion data can eliminate the need to perform complex perfusion image processing, including deconvolution with an arterial input function (AIF). In theory, deconvolution is a necessary step that corrects the concentration-time curves for confounding factors such as the rate and amount of contrast injection and the patient’s unique cardiac output function (Fieselmann et al., 2011). In practice, however, deconvolution is not as straightforward as it sounds, as it is highly dependent on the selection of the arterial input function (AIF) and more generally is an ill-posed problem that requires considerable regularization. This is likely the reason why it has been suggested that deep learning models using source CTP data may outperform those using deconvolved data (Robben et al., 2020; Amador et al., 2022). As of yet, the predictive value of conventional perfusion parameters and temporal features optimized using deep convolutional networks (with and without deconvolution) has not been formally compared for the task of lesion outcome prediction. Understanding how to use perfusion data optimally for deep learning remains an important consideration for developing state-of-the-art tissue outcome prediction models and, ultimately, improving the management of AIS.

Therefore, the aims of this study were to: (1) validate that deep learning provides the best results for predicting tissue outcomes from the acute CT perfusion images of ischemic stroke patients compared to classical machine learning models, and (2) compare tissue outcome predictions using perfusion parameter maps and raw perfusion data before and after deconvolution with an arterial input function.

## Materials and methods

### Patient data and image acquisition

Data was retrospectively collected from 145 acute ischemic stroke patients recruited for the ERASER thrombectomy study (Fiehler et al., 2019) or otherwise treated at the University Medical Center Hamburg-Eppendorf, Germany, from 2015 to 2019. The inclusion criteria were: (1) unilateral ischemic stroke due to anterior large vessel occlusion with a known time of symptom onset; (2) baseline CTP imaging acquired within 12 h of stroke symptom onset; (3) follow-up non-contrast CT (NCCT) imaging acquired within 72 h of symptom onset; (4) treatment within 24 h of symptom onset comprising either intravenous thrombolysis alone (IV; *n* = 43) or intra-arterial mechanical thrombectomy (IA; *n* = 102) with or without combined IV; and (5) absence of hemorrhage and prior stroke. Baseline and follow-up imaging were collected with voxel sizes of 0.45 × 0.45 × 5 mm and, for the baseline CTP, a temporal resolution of 1.6–1.8 s. Patient characteristics are presented in Table 1.

**TABLE 1:** Patient characteristics.

	All patients	IA patients	IV patients	*p* (IA vs. IV)
*N*, number of patients	145	102 (70%)	43 (30%)	–
Mean age (SD), years	71.18 (12.14)	70.61 (12.41)	72.53 (11.50)	0.385
Median NIHSS (IQR)	16 (6)	16 (6)	17 (6)	0.425
Sex, % female	43.45	49.02	30.23	0.037*
Median symptom onset to imaging (IQR), minutes	172 (146)	172 (140)	118 (162)	0.081
Mean ground truth lesion volume (SD), ml	74.59 (100.7)	55.64 (74.37)	117.5 (134.9)	0.005*
Successful reperfusion (TICI ≥ 2B)	–	78%	–	–
Complete reperfusion (TICI = 3)	–	26%	–	–

IA indicates treatment with intra-arterial mechanical thrombectomy with or without thrombolysis, while IV indicates treatment with thrombolysis only.

*Indicates statistical significance at alpha ≤ 0.05.

### Image processing

#### Admission computed tomography perfusion analysis

Patients’ acute CTP imaging was processed using the AnToNIa software (Forkert et al., 2014). First, the temporal mean intensity projection of the first three timepoints was computed, which we refer to as the baseline average image. Second, patient motion was corrected using 3D rigid registrations minimizing the mean squared difference between each timepoint of the CTP data and the baseline average image. Third, a baseline signal correction was performed by subtracting the baseline average image from each timepoint. Finally, the temporal resolution of the data was interpolated to one second and smoothed using a B-spline approximation. The data at this point in processing is referred to as the concentration-time curves (CTC) in the following.

The CTCs were deconvolved with an automatically computed arterial input function (Winder et al., 2020) using block-circulant singular value decomposition and a truncation threshold of 15% (Kjølby et al., 2006). The resulting residual curves (RC) were analyzed to generate perfusion parameter maps of cerebral blood flow (CBF), cerebral blood volume (CBV), mean transit time (MTT), and time-to-maximum (Tmax) using standard equations detailed in a previous work (Forkert et al., 2014). Additionally, a brain tissue mask was generated from the baseline average image by applying the following automatic segmentation pipeline: Gaussian blurring, binary thresholding in the range (1, 100) HU, erosion by a 5 × 5 × 1 kernel, connected component analysis keeping only the largest connected component, and then dilation by a 5 × 5 × 1 kernel. This mask was further refined into separate left- and right- hemisphere masks using a published method to define the hemispheric fissure (Forkert et al., 2014).

#### Follow-up non-contrast computed tomography analysis and registration

The tissue infarct on each follow-up NCCT scan was segmented using a semi-automatic region growing approach with the AnToNIa (Forkert et al., 2014) and ITK-SNAP (Yushkevich et al., 2006) software tools. This task was performed by a domain expert with more than a decade of experience in stroke image analysis. The follow-up infarct segmentation was aligned to the processed CTP imaging using a rigid transformation, which was computed by registering the NCCT image to the baseline average image using the SimpleITK (Beare et al., 2018) and ANTs (Avants et al., 2011) software packages. Finally, to correct for changes in CSF distribution due to swelling, the follow-up infarct segmentation was masked with the acute brain tissue mask.

#### Data preparation for machine learning

Each of the datasets used for model training and evaluation [concentration-time curves (CTC), deconvolved residual curves (RC), deconvolution-based perfusion parameters, and the ground-truth lesion] were masked using the ipsilateral hemisphere mask and then resampled to an in-slice spatial resolution of 0.45 × 0.45 mm^2^. To accommodate model operations requiring a fixed input shape, the resulting single-hemisphere images were padded to an in-slice size of 480 × 320 pixels and, for CTC and RC data, cropped to a 32-timepoint region of interest. For the CTC data, the temporal region of interest was calculated on a per-patient basis as follows: First, the contrast arrival time was estimated by selecting the first timepoint at which the mean enhancement (of all voxels within the brain tissue mask) was greater than 115% of the baseline image enhancement. Next, the temporal region of interest was centered on the timepoint with the maximum mean image intensity within the 40 s following contrast arrival. The RC, for which there was no global delay associated with the arrival of the contrast bolus (due to the correction with the arterial input function), were simply cropped to the first 32 timepoints. In all cases where the temporal region of interest extended beyond the beginning or end of the image, each curve was padded to the necessary length using the mean of the first three or last three existing timepoints, respectively.

For model training, each feature was independently re-centered and re-scaled to have a mean value of zero and a standard deviation of one, computed across the entire training data (including all timepoints for spatiotemporal features) for that model. For model testing, features were re-centered and rescaled using the same parameters that were applied to the model’s training data.

### Tissue outcome prediction methods

Multiple tissue outcome prediction methods were implemented and evaluated in this study. Although each method was unique with respect to its implementation details, which are described in the following subsections, all of the methods were evaluated according to a common process: model training, model testing, binarization, and statistical evaluation. The purpose of training was to optimize a model’s internal weights such that, given acute perfusion data for one or more voxels, the model would assign each voxel a value in the continuous range [0, 1] corresponding as closely as possible to the voxel’s known tissue outcome of either infarct (1) or non-infarct (0). During testing, patient datasets not used during training were analyzed in their entirety by the trained model to generate a 3D map of probabilistic tissue outcomes for each patient. In the binarization step, each of these probabilistic 3D maps was thresholded to create a binary (infarct/non-infarct) lesion segmentation that could be compared directly to the ground-truth lesion segmentation in the final statistical evaluation step. For Tmax thresholding, which is not a machine learning method and has no associated model, patients’ Tmax maps were used in lieu of the maps typically produced by model testing and threshold optimization was performed as described below.

**FIGURE 1:**
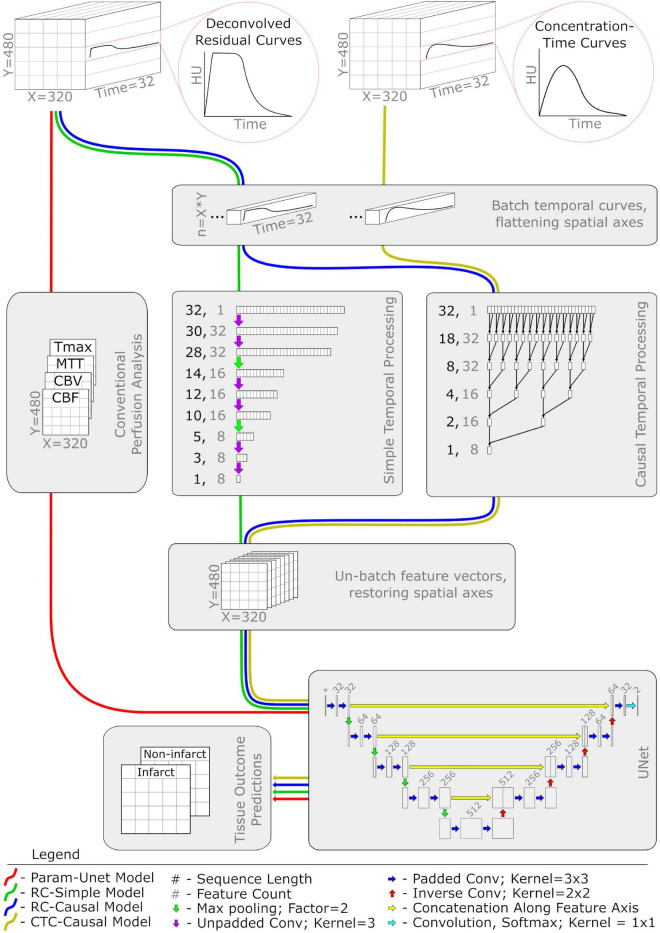
Network diagram of the four deep learning classifiers used in this study. Each colored pathway illustrates the flow of information for a different model. Abbreviations and acronyms are as follows: CTP, computed tomography perfusion imaging; CBF, cerebral blood flow; CBV, cerebral blood volume; MTT, mean transit time; Tmax, time to maximum of the residual curve; conv, convolution.

The different tissue outcome prediction models were separately applied and evaluated using only the patients treated with intra-arterial mechanical thrombectomy (IA), and then using only the patients treated with intravenous thrombolysis (IV). This approach guarantees functionally independent predictive models for different treatment approaches, which is important for many potential applications of tissue outcome prediction such as treatment efficacy analysis (Fiehler et al., 2019; Winder et al., 2021). The deep learning models are also visualized in Figure 1.

#### Optimal time-to-maximum thresholding

The first approach evaluated in this work was a simple Tmax thresholding that was meant to serve as a clinically easy-to-implement baseline comparison method. More precisely, voxels were predicted as infarct only if their Tmax exceeded a given threshold value. To determine which threshold value produces the most accurate segmentations of the ground-truth infarct, 120 different threshold values ranging from 0 to 24 s at an interval of 0.2 s were used to binarize every patient’s Tmax map. To avoid using knowledge of a patient’s ground truth infarct to optimize the binary thresholding of their own Tmax map, we employed a leave-one-out approach. In essence, for each patient, the mean Dice similarity metric comparing the binarized Tmax segmentation to the ground-truth infarct was computed over all other patients from the same treatment group at each of the 120 threshold values. The threshold value that maximized the leave-one-out Dice value for each patient was then used to binarize the patient-individual Tmax map, producing the final tissue outcome prediction.

#### Random decision forest

A random forest classifier representing a classical machine learning approach served as a secondary comparison method. The classifier was implemented using the random decision forest (RDF) regressor distributed in the alglib^1^ software package. Each forest comprised 100 trees and the single hyperparameter *r* (the proportion of the total training instances used to fit an individual tree) was set to 0.5.

The input to the RDF consisted of the four perfusion parameters (CBF, CBV, MTT, and Tmax) associated with each individual voxel sampled from within the ipsilateral hemisphere mask. To balance the class labels in the training data, we selected voxels for training on a per-patient basis using stratified random undersampling, whereby all voxels from the minority class (typically infarct) were sampled alongside an equal number of randomly selected voxels from the majority class (typically non-infarct). The importance of this step is more thoroughly described in a previous publication (Winder et al., 2019). Previous studies also suggest that, because the RDF does not consider a voxel’s spatial context, the resulting binary infarct segmentations are prone to noise and typically benefit from additional filtering steps (Winder et al., 2019). Accordingly, we applied in-slice morphological closing using a 3 × 3 voxel kernel followed by a connected component analysis to remove any components consisting of less than 10 voxels before continuing with any other processing. To justify including this additional filtering step only for the RDF model, we also compared each model with and without noise-reduction filtering as reported in Section “Applying filters for noise reduction” below.

#### UNet with perfusion parameters (Param-UNet)

As a deep learning alternative to the random forest model, we constructed a 2D UNet (Ronneberger et al., 2015) with minor modifications (see Figure 1). The input to the model consisted of the four perfusion parameter maps (CBF, CBV, MTT, and Tmax) representing a single image slice. The intermediate layers were implemented according to the original publication (Ronneberger et al., 2015) with the following exceptions: at each convolutional layer, zero-padded convolutions were used to maintain a consistent image size and a weak L2 kernel regularizer (having a factor of 0.5e-4) was used to discourage overfitting. The terminal layer, a 1 × 1 convolution with softmax activation, structured the output of the model as a map of probabilistic voxel-wise tissue outcomes with dimensions equal to the input image slice.

Model weights were trained using the Adam optimizer (α = 0.0005; β1 = 0.975; β2 = 0.999; ε = 0.08) (Kingma and Ba, 2017) and the soft Dice loss function. Specifically for training, image slices that did not have any infarct voxels in their corresponding ground-truth lesion segmentation were omitted, as the Dice loss function is not mathematically defined in this case. In the following, we refer to models using this architecture as Param-UNet.

#### UNet with residual curves (RC-Simple, RC-Causal)

To investigate whether the perfusion parameters typically computed and used in the clinical routine contain all predictive temporal information that is measured by CTP datasets for machine learning, we implemented additional models that are able to learn temporal features directly from the deconvolved residual curves (RC). Compared to the Param-UNet model, these models replaced the input layer corresponding to the perfusion parameter maps with a temporal processing block (Figure 1) that acted as a trainable feature extractor over the RC data. Otherwise, the models were identical.

Within the temporal processing block, each voxel’s RC was independently processed using one of the two 1D convolutional architectures evaluated in this work. Both architectures reduced each RC, originally having 32 timepoints, to a set of eight temporal features (a feature vector). Spatial maps of resulting feature vectors (analogous in structure to the typical perfusion parameter maps CBF, CBV, MTT, and Tmax) were then provided as input features to the UNet. The temporal processing blocks were trained in tandem with the UNet, each having a single set of weights optimized over all RCs present in the model’s training data.

The first convolutional architecture used for the temporal processing block was inspired by the structure of the UNet. It contains three pairs of 1D convolutional layers with a max pooling layer using a factor of two between each pair. Each convolutional layer was configured to use un-padded convolutions with a kernel width of 3 and ReLu activation. The number of filters for each pair of convolutional layers is 32, 16, and 8, respectively, which results in the output of the final convolutional layer generating eight temporal features (Figure 1). In the following, we refer to models using this architecture as RC-Simple.

Although UNet-inspired architectures are well-validated and widely used for computer vision problems, they are generally used to interpret spatial rather than temporal data. There is evidence that data representing temporal sequences may be better analyzed using more specialized architectures such as dilated causal convolutions (van den Oord et al., 2016), especially for feature extraction within a larger spatiotemporal model (Castro et al., 2021). Thus, the second convolutional architecture used for the temporal processing block was a sequence of five 1D causal convolutional layers. These layers were configured with 8 filters, a kernel width of 2, ReLu activation, and dilation rates corresponding to increasing powers of 2 with network depth (1, 2, 4, 8, and 16). For 1D sequences, causal convolution is functionally equivalent to normal convolution except that the output is shifted so that each of its elements depends only on inputs corresponding to earlier timepoints. Because of this property, only the last element in the output of the fifth causal convolutional layer combines information from all 32 timepoints of the input RC. This last element, a vector of eight features computed from the entire RC, was therefore isolated as the output of the temporal processing block (Figure 1). In the following, we refer to models using this architecture as RC-Causal.

#### UNet with concentration time curves (CTC-Causal)

In theory, RCs should be better suited for tissue outcome prediction because unwanted signal caused by inter-patient differences related to the contrast injection protocol and cardiac output function is removed by deconvolution with the arterial input function (AIF) (Fieselmann et al., 2011). However, as previously mentioned, deconvolution itself is an ill-posed problem and can be highly dependent on the AIF definition. To investigate the reported effects of deconvolution, we trained models identical in structure and function to the RC-Causal model, only using the cropped and centered CTCs instead of the deconvolved RCs as input. We will refer to these models as CTC-Causal in the following.

### Model evaluation

Within each treatment group, a patient-level fivefold cross-validation scheme was used to allow for the prediction of every patient’s follow-up infarct segmentation while still maintaining independent training and testing sets. The folds for this cross-validation approach were assigned once and applied consistently across every tissue outcome prediction method to allow for a fair comparison of the results. For training the deep learning models (Param-UNet, RC-Simple, RC-Causal, and CTC-Causal), a validation set was constructed by sampling patients from the training set, resulting in a final data allocation of 68% for training, 12% for validation, and 20% for testing. To prevent overfitting, training was terminated after the validation loss failed to improve for eight consecutive epochs. At this point, the model weights corresponding to the epoch with the lowest validation loss were restored.

For the RDF and each of the deep-learning based methods, the optimal threshold for binarizing the tissue outcome prediction maps may differ by model (i.e., 0.5 may not be the best threshold in all cases) (Guo et al., 2017). To determine the optimal threshold, each of a model’s tissue outcome predictions was binarized at thresholds ranging from 0 to 1 using intervals of 0.01 and compared to the corresponding ground-truth tissue infarct using the Dice metric. The optimal threshold was then calculated using the same leave-one-out optimization method described for Tmax thresholding in Section “Optimal time-to-maximum thresholding.”

For all of the implemented tissue outcome prediction methods, the Dice value comparing each predicted lesion segmentation (binarized at its optimal threshold) to its corresponding ground-truth infarct was recorded as a measure of the model’s performance. Additionally, we computed the area under the receiver operating characteristic curve (ROC-AUC) as a threshold-independent performance metric and the volume error of the binarized prediction as a metric of particular interest to clinicians. For each method, each patient’s empirical ROC curve was constructed using the voxel-wise false positive rate and mean true positive rate computed between the ground-truth and predicted lesions binarized about a series of 100 threshold values. The final ROC curve for each method was computed as the average of the corresponding individual patient curves.

### Statistical analysis

All statistical tests were performed in IBM SPSS 25 using an alpha of 0.05 to define significant results. Patient characteristics were compared between the IA and IV patient groups using an independent samples *t*-test, Mann–Whitney *U* test, or chi-squared test, as appropriate.

For each tissue outcome prediction method, the Dice values obtained with and without applying the noise-reduction filtering described in Section “Random decision forest” were compared using a paired *t*-test and the Sidak correction to maintain a family-wise error rate of 0.05.

The tissue outcome prediction methods were statistically compared using the Dice values, ROC-AUC, volume errors, and absolute volume errors of their predictions (pooled between the IA and IV patient groups) using repeated-measures ANOVAs. For each outcome measure, Mauchly’s sphericity test was performed and, if significant, the Greenhouse-Geisser correction was applied to the corresponding ANOVA. In the event of a significant ANOVA, *post-hoc* pairwise comparisons using the Sidak correction were also performed.

For each deep learning method capable of learning temporal features (RC-Simple, RC-Causal, CTC-Causal), maps of the eight convolutionally optimized temporal features were correlated to each of the four perfusion parameter maps using Spearman’s rank correlation coefficient. Spearman’s rho values reported in this study were computed from the pooled foreground voxels of all testing datasets (from both the IA and IV treatment groups).

## Results

### Patient characteristics

Descriptive statistics for the patient sample used in this work, both pooled and by patient group, are shown in Table 1. The prevalence of female patients was significantly greater in the IA patient group (49%) compared to the IV patient group (30%; *p* = 0.037). However, no significant differences were identified between the groups with respect to age, NIHSS at admission, or symptom onset to imaging times. The optimal Tmax threshold for both patient groups was independently determined to be 7.2 s.

### Applying filters for noise reduction

The mean Dice values for each model with and without the noise-reduction operations described in Section “Random decision forest” are displayed in Table 2. Noise-reduction significantly improved the Dice values observed from the RDF method [95% Confidence Interval of the difference (CID) = 0.022–0.037; *p* < 0.001]. For the other methods, however, the effect of this was either insignificant (Tmax thresholding: *p* = 0.861; CTC-Causal: *p* = 0.301) or of negligible effect size (Param U-Net: CID = −0.002 to −0.005, *p* < 0.001; RC-Simple: CID = 0.000 to 0.002, *p* < 0.001; RC-Causal: CID = 0.000 to 0.002, *p* = 0.034). Accordingly, the following statistics are reported for the RDF with noise reduction and all other models without.

**TABLE 2:** Mean Dice value for each tissue outcome prediction method with and without applying additional filtering operations to each tissue outcome prediction for the purpose of noise reduction.

	Tmax thresholding	Random decision forest	Param-UNet	RC-Simple	RC-Causal	CTC-Causal
Noise-removal post-processing	0.248 (0.217)	**0.262 (0.213)**	0.284 (0.229)	0.277 (0.228)	0.286 (0.229)	0.297 (0.235)
No noise-removal post-processing	**0.249 (0.214)**	0.233 (0.199)	**0.287 (0.229)**	**0.276 (0.228)**	**0.286 (0.228)**	**0.296 (0.234)**

Bold cells indicate the models chosen to represent each architecture in subsequent analyses.

Abbreviated model names correspond to: Tmax thresholding, random decision forests (RDF), deep learning from perfusion parameter maps (Param-UNet), deep learning from deconvolved residual curves with convolutional (RC-Simple) or causal convolutional (RC-Causal) feature extraction, and deep learning from source concentration-time curves (CTC-Causal).

### Comparing tissue outcome prediction methods

**FIGURE 2:**
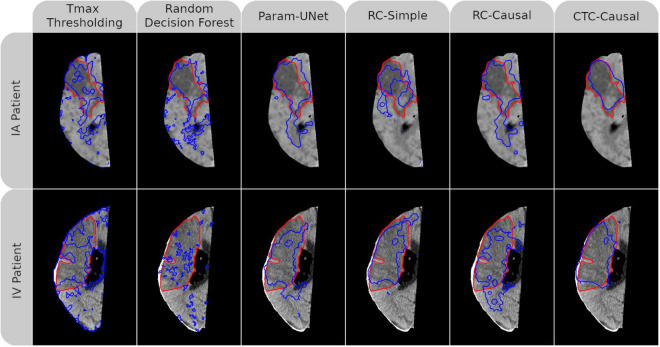
Follow-up non-contrast CT imaging with superimposed ground-truth lesion segmentation (red) and tissue outcome prediction (blue). An AIS patient treated with intraarterial mechanical thrombectomy (IA) is shown in the top row while another treated with intravenous tPA (IV) is shown in the bottom row. A different tissue outcome prediction method is shown in each column. For both patients, the individual slice with the greatest cross-sectional ground truth area is shown. Dice values for each (3D) tissue outcome prediction are, clockwise from the top-left, 0.45, 0.40, 0.54, 0.20, 0.48, 0.75, 0.73, 0.56, 0.70, 0.60, 0.24, and 0.53. Abbreviated model names correspond to: Tmax thresholding, random decision forests (RDF), deep learning from perfusion parameter maps (Param-UNet), deep learning from deconvolved residual curves with convolutional (RC-Simple) or causal convolutional (RC-Causal) feature extraction, and deep learning from source concentration-time curves (CTC-Causal).

Mean values for the Dice, ROC-AUC, and infarct volume errors for each tissue outcome prediction method are presented in Table 3. The methods differed significantly with respect to the Dice values of their predictions (*p* < 0.001) with all deep learning models outperforming Tmax thresholding (RC-Simple: *p* = 0.034; RC-Causal, CTC-Causal, and Param-UNet: *p* < 0.001). The top-performing deep learning models, Param-UNet and CTC-Causal, also outperformed the random decision forest (*p* < 0.001 and *p* = 0.022, respectively). The CTC-Causal model led to the best mean Dice (0.296) but this difference was not statistically significant compared to other deep learning models. The binarized lesion predictions generated by each tissue outcome prediction method, for a patient from each treatment group, are shown in Figure 2. Notable qualitative observations include the greater spatial coherence of the deep learning methods compared to Tmax thresholding and the RDF, as well as an apparently greater specificity for the CTC-Causal model compared to the RC-Causal model.

**TABLE 3:** Mean values and (standard deviations) for the Dice values, area under the ROC curve (AUC), volume errors, and absolute volume errors of each tissue outcome prediction method.

		Total	IA patients	IV patients
Dice	Tmax thresholding	0.249 (0.214)	0.217 (0.204)	0.324 (0.220)
	Random decision forest	0.262 (0.213)	0.206 (0.188)	0.300 (0.211)
	Param-UNet	0.287 (0.229)	0.252 (0.217)	0.369 (0.240)
	RC-Simple	0.276 (0.232)	0.259 (0.220)	0.314 (0.244)
	RC-Causal	0.286 (0.228)	0.260 (0.219)	0.346 (0.240)
	CTC-Causal	**0.296 (0.234)**	**0.264 (0.211)**	**0.384 (0.264)**
ROC-AUC	Tmax thresholding	0.693 (0.320)	0.690 (0.301)	0.698 (0.362)
	Random decision forest	0.740 (0.125)	0.741 (0.120)	0.737 (0.140)
	Param-UNet	0.773 (0.146)	0.781 (0.147)	0.768 (0.144)
	RC-Simple	0.764 (0.184)	0.770 (0.186)	0.767 (0.182)
	RC-Causal	0.768 (0.153)	0.783 (0.147)	0.747 (0.167)
	CTC-Causal	**0.791 (0.142)**	**0.786 (0.133)**	**0.802 (0.159)**
Volume error (ml)	Tmax thresholding	83.8 (95.1)	93.2 (85.1)	61.42 (113.54)
	Random decision forest	**48.2 (98.5)**	48.8 (83.5)	46.5 (128.5)
	Param-UNet	53.6 (100.0)	53.8 (88.1)	53.1 (124.9)
	RC-Simple	66.4 (141.8)	48.1 (82.1)	109.9 (223.5)
	RC-Causal	58.8 (122.4)	**36.5 (85.5)**	111.8 (172.4)
	CTC-Causal	48.5 (93.6)	50.1 (89.0)	**44.7 (104.8)**
Abs. volume error (ml)	Tmax thresholding	107.8 (66.4)	108.6 (64.1)	106.0 (72.4)
	Random decision forest	86.6 (66.9)	76.4 (59.0)	110.9 (78.4)
	Param-UNet	89.1 (70.1)	81.2 (63.6)	107.8 (81.3)
	RC-Simple	106.3 (114.7)	72.4 (61.5)	186.8 (163.1)
	RC-Causal	97.1 (94.8)	**68.4 (62.7)**	165.2 (120.8)
	CTC-Causal	**77.4 (71.5)**	76.7 (67.1)	**78.8 (81.7)**

IA indicates treatment with intra-arterial mechanical thrombectomy with or without thrombolysis, while IV indicates treatment with thrombolysis only.

The values corresponding to the best model performance for each performance metric are shown in bold.

Abbreviated model names correspond to: Tmax thresholding, random decision forests (RDF), deep learning from perfusion parameter maps (Param-UNet), deep learning from deconvolved residual curves with convolutional (RC-Simple) or causal convolutional (RC-Causal) feature extraction, and deep learning from source concentration-time curves (CTC-Causal).

**FIGURE 3:**
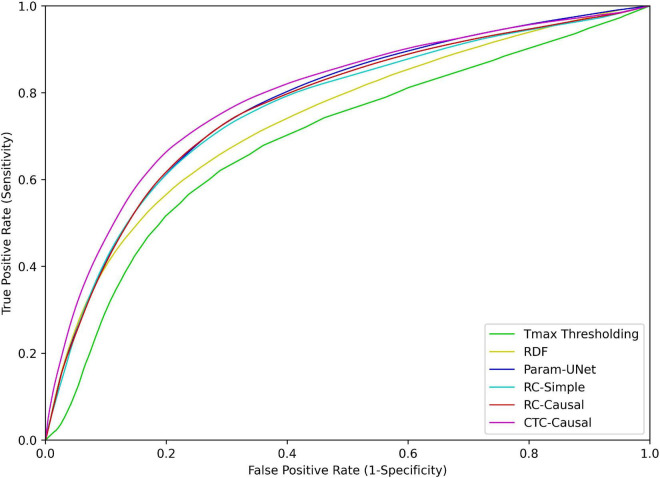
Receiver operating characteristic (ROC) curves for each of the tissue outcome prediction methods. Abbreviated model names correspond to: Tmax thresholding, random decision forests (RDF), deep learning from perfusion parameter maps (Param-UNet), deep learning from deconvolved residual curves with convolutional (RC-Simple) or causal convolutional (RC-Causal) feature extraction, and deep learning from source concentration-time curves (CTC-Causal).

The methods also differed significantly with respect to the ROC-AUC values (*p* < 0.001). Similar to the trend observed for the Dice values, Tmax thresholding produced significantly lower ROC-AUC values compared to the RDF (*p* < 0.001), which in turn produced lower ROC-AUC values compared to any of the deep learning models (*p* < 0.001 in all cases). However, there were no significant differences between the deep learning models in this respect. The ROC curves for each tissue outcome prediction method are shown in Figure 3.

**FIGURE 4:**
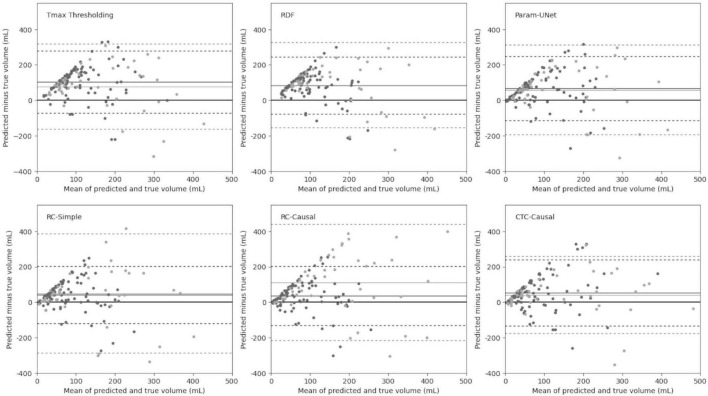
Bland–Altman plots for the predicted infarct volumes of each tissue outcome prediction method. Dark markers and lines represent patients treated with intravenous tPA (IV) alone, while light markers and lines represent patients treated with intra-arterial mechanical thrombectomy (IA) with or without combined IV. Abbreviated model names correspond to: Tmax thresholding, random decision forests (RDF), deep learning from perfusion parameter maps (Param-UNet), deep learning from deconvolved residual curves with convolutional (RC-Simple) or causal convolutional (RC-Causal) feature extraction, and deep learning from source concentration-time curves (CTC-Causal).

Finally, the volume error and absolute volume error also differed significantly between models (*p* < 0.001 in both cases). Although all tissue outcome prediction methods tended to overestimate the follow-up infarct volume based on the mean volume error, Tmax thresholding overestimated the infarct volumes by the greatest margin (pairwise comparisons to RC-Simple: *p* = 0.712; RC-Causal: *p* = 0.016; RDF, Param-UNet, and CTC-Causal: *p* < 0.001). Tmax thresholding also produced the greatest mean absolute volume error, which was statistically significant in comparison to the RDF, Param-UNet, and CTC-Causal models (*p* < 0.001). The CTC-Causal model produced small mean absolute volume errors compared to the RC-Simple model, but once again did not reach statistical significance (*p* = 0.057). Bland–Altman plots of the predicted lesion volumes for each model are shown in Figure 4.

For the deep learning models that derived their own temporal features (RC-Simple, RC-Causal, and CTC-Causal), the correlations between their unique feature maps and each of the four conventional perfusion parameter maps (CBF, MBV, MTT, Tmax) are shown in the Supplementary Figure 1. All correlations shown are statistically significant with *p* < 0.001. The feature maps for the CTC-Causal model are visualized in Supplementary Figure 2 for the same two patients previously shown in Figure 2. Notably, models that utilize residual curve data (RC-Simple, RC-Causal) derive several features directly correlated to CBF/CBV and inversely correlated to MTT/Tmax (or vice-versa), which corresponds with the known profile of cerebral infarction (Demeestere et al., 2020). While many features learned directly from CTC bear a visual resemblance to conventional perfusion parameters, they show noticeably weaker correlations compared to features derived from residual curves, which suggests a unique organization of the relevant temporal information.

## Discussion

The main finding of this work was that the deep learning model that automatically derived temporal features directly from the concentration-time curves of patients’ acute CTP imaging (CTC-Causal) produced the best Dice values and absolute volume errors. Considering all metrics, the machine learning models using the raw concentration-time curves for tissue outcome prediction trended toward better results compared to using deconvolved CTP datasets (RC-Causal and RC-Simple) despite not reaching statistical significance. Additionally, tissue outcome predictions computed using deconvolution-based perfusion parameter maps (Param-UNet) were not significantly better than any made from learned temporal features. All deep learning methods outperformed the random decision forest, which in turn outperformed simple Tmax thresholding, by measure of the methods’ ROC-AUC values.

### Performance of deep learning

Although several deep learning methods for stroke tissue outcome prediction have already been published (Nielsen et al., 2018; Winzeck et al., 2018; Ho et al., 2019; Robben et al., 2020; Yu et al., 2020; Amador et al., 2022), this is among the first to demonstrate their state-of-the-art performance compared to the leading methods currently employed in both research (decision forests) and clinical practice (Tmax thresholding). Our observation that the CTC-Causal and Param-UNet deep learning models both produced better Dice and ROC-AUC values than either of the previously mentioned comparison methods indicates that deep learning may indeed play an important role in the future management of AIS by enabling researchers and clinicians to make more accurate tissue outcome predictions. Furthermore, the approximately equivalent performance of the CTC-Causal and Param-UNet models suggests that deep learning could also be used to improve the speed and objectivity of tissue outcome prediction by eliminating the need for AIF selection and deconvolution. The advantages of deep learning are not as immediately obvious from the observed volume errors, which must be interpreted with the understanding that the predicted lesion volume does not consider the location of the predicted lesion. In the most extreme case, a model could predict the correct number of infarct voxels entirely outside the ground-truth lesion segmentation, producing both a Dice value and a volume error of zero. As a result, the clinical utility of a method must be interpreted considering both the volume error and Dice or ROC-AUC in conjunction. Although the RDF trended toward the lowest volume errors, its relatively worse performance compared to deep learning for all other metrics suggests that deep learning is overall more performant.

Comparing the predictive accuracy of the tissue outcome prediction methods in this study to previously published studies is complicated by a number of factors. Specifically, the performance of a model depends not only on its architecture, but also on the data used for training and testing as well as the performance metric being evaluated. In the context of the Dice metric, for example, it is much easier to obtain a high Dice value from large ground truth lesions compared to small lesions. This may partially explain the observed trend, albeit not statistically significant, toward better Dice values for the IV cohort compared to the IA cohort. Additionally, lower performance metrics are expected for datasets with greater unexplained variance. Possible sources of unexplained variance in the current study include differences in image acquisition hardware and protocol between medical centers, the time elapsed between acute and follow-up imaging, patients’ acute infarct volume and collateral status, and the time and rate of successful or complete reperfusion following treatment. Without controlling for such factors, the performance metrics published in different tissue outcome prediction studies are not directly comparable. The free and easily accessible RDF implementation distributed with the alglib software library could act as a common point of comparison between tissue outcome prediction studies, providing some insight into the difficulty of the stroke tissue outcome prediction problem based on the datasets used. Therefore, although many previous deep learning studies do report better performance metrics compared to the current study, we are confident that this disparity is not due to serious errors or flaws in the design of the proposed deep learning methods since the Param-UNet and CTC-Causal models significantly outperformed the RDF according to every performance metric when the training and testing data was controlled.

### Effects of deconvolution and auxiliary inputs

Considering the widely acknowledged importance of deconvolution in clinical perfusion image analysis (Fieselmann et al., 2011), it is somewhat counterintuitive that the highest Dice values observed in this study were produced by the CTC-Causal model. To date, few other studies have attempted to predict stroke tissue outcomes using features extracted directly from a patient’s non-deconvolved concentration-time curves (CTC). However, the findings of these studies generally agree with the current results. For example, Ho et al. (2019) proposed a deep convolutional neural network for stroke outcome prediction using CTCs from MR perfusion imaging. Although Ho et al. (2019) used regression and instance-based methods for comparison while the current study used random decision forests, both studies found that the CTC-based deep learning model produced significantly higher ROC-AUC values than their respective comparison methods trained using perfusion parameter maps. From their experiment, Ho et al. (2019) concluded that deep learning models that use CTC data benefit from both the ability to perform a deconvolution-free analysis and the ability to learn temporal features much greater in complexity than traditional perfusion parameters. However, Ho et al. (2019) did not train equivalent deep learning models using residual curves (RCs) or deconvolved perfusion parameters to fully justify these claims. Although the current study’s CTC-Causal model produced greater Dice values than the RC-Causal model, which supports the notion that there is a benefit to deconvolution-free analysis, the relatively similar performance of the Param-UNet, RC-Causal, and RC-Simple models suggests that a more complex analysis of the RCs does not necessarily lead to temporal features that are more predictive than the perfusion parameter maps.

In a CT perfusion study, Robben et al. (2020) specifically compared CTCs and deconvolved RCs as the input to a 3D deep convolutional neural network for predicting stroke tissue outcomes. Robben et al. (2020) found that their model produced significantly greater Dice values using the CTCs as compared to the RCs. However, rather than considering their CTC-based model a form of deconvolution-free analysis, Robben et al. (2020) included the arterial input function (AIF) as an input feature and hypothesized that their model was able to perform an implicit deconvolution of the CTC data (although this was never fully evaluated). Because the differences between the CTC-Causal and RC-Causal models of the current study did not reach statistical significance, the results of Robben et al. (2020) raise the question of whether CTC-based deep learning methods require knowledge of the AIF, or some other means of controlling for inter-patient variability, to significantly outperform equivalent models based on RC data.

Therefore, it remains to be determined how including information about the contralateral hemisphere, the AIF, or potentially a combination of the two affects the performance of these CTC-based deep learning models. In a similar vein, patient-specific clinical factors such as age, sex, baseline NIHSS score and ASPECTS, rtPA administration, and hyperglycemia (Eilaghi et al., 2014), dehydration (Bahouth et al., 2018), time-to-scan and time-to-treatment (d’Esterre et al., 2015), collateral status (Ernst et al., 2015), as well as tissue specific factors such as distance to the ischemic core (Winder et al., 2019), brain region (Payabvash et al., 2011), and myelination (Chen et al., 2019) are all known to affect the tissue’s vulnerability to infarction. Many machine learning models combine this tabular data with imaging features to improve the accuracy of the resulting tissue outcome predictions (Winder et al., 2019; Robben et al., 2020). At present, however, the question of which non-imaging features to include and how best to integrate them does not seem to have a definite answer, especially for deep learning methods. Nevertheless, we believe that including clinical parameters will likely benefit all models proportionally such that the main findings of this work still hold true.

### Current and future applications of machine learning-based stroke tissue outcome prediction

An ensemble of machine learning-based stroke tissue outcome prediction methods can be used to model the evolution of an acute ischemic stroke under multiple different treatment conditions. Applied to a population of patients, such an ensemble of machine learning models trained on a rather small set of datasets could help to determine if a novel treatment devices or therapy has the potential for a full-scale clinical trial based on its predicted tissue salvage. This is referred to as *in silico* treatment efficacy analysis (Winder et al., 2021). Applied to individual patients, a similar ensemble could help clinicians to understand how a particular case of stroke will evolve without medical intervention and then balance the potential tissue salvage of different treatment options with their respective financial costs and health risks. These two potential applications of stroke tissue outcome prediction are alike in that both require highly accurate predictive algorithms, but they are also quite distinct in terms of the practical and administrative barriers that must be overcome to use them in the clinical setting.

Health Canada and the U.S. Food and Drug administration (FDA) regulate which medical software can be used directly in the clinical environment, but neither entity currently provides a framework for the approval of continuously learning artificial intelligence methods. As a result, machine learning-based stroke tissue outcome prediction has, historically, only been applied to *in silico* treatment efficacy analysis for pre-clinical pilot studies (Fiehler et al., 2019). In this context, considering the rapid development of novel thrombectomy devices (Munoz et al., 2022) and the large number of proposed neuroprotectant drugs (Frank et al., 2022), there is inherent value in the development of reliable tissue outcome prediction pipelines that can be constructed quickly with minimal manual processing. Additionally, CTP imaging is significantly more accessible compared to perfusion-weighted MRI in North America (Demeestere et al., 2020), but also more difficult to process due to its worse signal-to-noise ratio, making tissue outcome prediction from CTP data an important research topic. For these reasons, a valuable future study may be to validate whether the proposed CTC-Causal architecture is viable for *in silico* treatment efficacy analysis or requires further development. Alternatively, in the context of tissue outcome prediction as a clinical decision aid, it is important to note that the FDA is currently collaborating with researchers and clinicians to develop a framework for the approval of artificial intelligence and machine learning-based software (US Food and Drug Administration [FDA], 2021). Elements of software development such as data preprocessing and classifier design are important considerations of this collaborative effort. Therefore, the current value of the proposed deep learning architecture is not that it can be applied to clinical tasks in its existing state, but rather that it provides a way to improve the accessibility of CTP-based stroke tissue outcome prediction and highlights the need to consider deep-learning based feature extraction in the emerging FDA regulatory guidelines.

In future studies, it should be investigated how best to integrate tabular and auxiliary spatial variables, including those listed in Section “Effects of deconvolution and auxiliary inputs,” into deep learning models. For those clinical variables which are derived from patients’ acute imaging, such as baseline ASPECTS and collateral status, there is the potential for deep learning to extract the information automatically. To explicitly enforce that a model learns these features, it would be possible to use a multi-output model that predicts both ASPECTS/collateral status and tissue outcome simultaneously. Similarly, relevant variables that are not normally known before treatment, such as reperfusion time, could be included as model input features to help clinicians plan for contingencies such as treatment delays or triage to primary care centers with thrombectomy resources (Kemmling et al., 2015). Overall, including these additional relevant variables in the tissue outcome prediction models is likely to improve their accuracy in predicting the final infarct, which is important for any prospective clinical application.

In addition to predicting the final infarct, stroke tissue outcome prediction methods could also theoretically be used to predict other clinically relevant stroke outcomes. For instance, predicted infarct segmentations could be combined with a lesion symptom mapping approach to predict patients’ specific functional deficits (for example, NIHSS sub-scores) from their acute imaging (Rajashekar et al., 2022). Using deep learning, a particular model trained to predict tissue outcomes could be extended to also predict functional outcomes, absorbing the role of lesion symptom mapping. The feasibility of this idea is supported in part by a recent pre-print that describes a deep learning model capable of reliably predicting patients’ ordinal mRS from tabular and diffusion-weighted MR imaging data (Herzog et al., 2022). Finally, stroke tissue outcome prediction models might be easily adapted to predict post-stroke complications such as life-threatening malignant edema. A large final infarct volume is itself a predictor of malignant edema, but malignant edema and ischemic infarct growth also share several common neuroimaging predictors, including poor collateral status and low venous blood flow (Zhang et al., 2022). Considering these similarities, extending a deep learning model trained for tissue prediction to also estimate the likelihood of malignant edema could be a valuable experiment.

### Limitations

There are several limitations of this study that should be highlighted. First, this study utilized retrospective patient data, meaning that it was not possible to exercise complete control over factors such as patient age, sex, and the time of their acute and follow-up imaging. Statistics for these factors are reported, if available, in Table 1 to indicate potential sources of variability and bias. Significant differences between the IA and IV cohorts do not introduce bias into a comparison of different model architectures, considering that each architecture was trained and evaluated on the same patient data. However, any comparison between one model trained on the IA cohort and another model trained on the IV cohort would be subject to this bias, which limits the applicability of the models in their current state to tasks such as *in silico* treatment efficacy analysis (Winder et al., 2021). Furthermore, our models are agnostic to potentially relevant clinical variables such as those listed at the end of Section “Effects of deconvolution and auxiliary inputs” and, as mentioned in Section “Current and future applications of machine learning-based stroke tissue outcome prediction,” further experimentation is required to integrate these variables.

Second, the results of this study are, strictly speaking, only valid for patients meeting our relatively restrictive inclusion criteria. Thus, it remains speculative if the results will hold true for patients experiencing posterior circulation ischemic stroke (20–25% of ischemic stroke) (Merwick and Werring, 2014), hemorrhagic transformation (∼8%) (Spronk et al., 2021), an unknown symptom onset time (14%) (Mackey et al., 2011), or a symptom onset to treatment time greater than 24 h. Among the patients with an unknown symptom onset time, those experiencing wake-up stroke have been shown to especially benefit from fast and correct CTP analysis (Ajčević et al., 2020), so that a future investigation concerning the reliability of stroke tissue outcome prediction for wake-up stroke is warranted.

Third, because this study utilized real medical images, neither a patient’s AIF nor the boundary of their follow-up infarct lesion could be determined with perfect accuracy. We attempted to mitigate sources of error related to the AIF determination by employing an automatic AIF definition method that consolidates hemodynamic information from multiple voxels to reduce noise while preserving important AIF shape characteristics (Winder et al., 2020). We also employed a semi-automatic region growing approach to reduce the subjectivity of the follow-up infarct segmentations. However, because of the coarse slice thickness of follow-up imaging (5 mm), the boundary of the infarct segmentation may not be ideal after resampling to the patient’s acute image space. One potential solution to address these inaccuracies would be to use fuzzy logic-based segmentation or image enhancement to refine the boundary of the infarct segmentation. Efficient so-called fuzzy image preprocessors have recently been proposed that run in real-time, adding negligible computational overhead to the image preprocessing pipeline, and have proven effective on difficult segmentation tasks related to diabetic retinopathy (Jamal et al., 2012; Versaci et al., 2015).

Fourth, all follow-up imaging was acquired within the first few days post-ictus, which roughly coincides with the period of greatest post-stroke inflammation (Lansberg et al., 2001). As a result, the follow-up segmentation may overestimate the final infarct volumes. However, neuroinflammation may also play an appreciable role the in pathogenesis of acute ischemic stroke, making the subacute ischemic lesion as valuable as a target for prediction as the chronic ischemic lesion (Pluta et al., 2021). Furthermore, ischemic stroke lesions typically continue to evolve even in the chronic phase, which makes restricting the time window for follow-up imaging to a narrow range of dates an important consideration for curating a dataset (Merwick and Werring, 2014; Goyal et al., 2020; Pluta et al., 2021). Considering that the range of dates for which follow-up imaging can be acquired in the chronic phase of ischemic stroke is typically very large, filtering a dataset to include only early follow-up imaging within a narrow time window may, in some cases, make it easier to train accurate machine learning models. Changes in cerebrospinal fluid (CSF) distribution due to edema between the time of acute and follow-up imaging may also affect the images’ co-registration, causing some of the follow-up infarct to eclipse regions of CSF on the acute imaging and, therefore, be impossible to predict as infarct. To mitigate this error for minor swelling, we masked the registered follow-up infarct segmentation with the acute brain tissue mask during our image preprocessing. Furthermore, no severe swelling or hemispheric midline shifts were observed on follow-up imaging.

Finally, the models developed in this study were validated internally using a fivefold cross validation due to the relatively small number of available patient datasets. However, an external validation would better represent the models’ performance in an applied clinical setting. Therefore, while the current study may be valuable in informing the design of future deep learning algorithms for stroke tissue outcome prediction, any such method that is developed for clinical use will require significantly more training data and rigorous external validation.

## Conclusion

Deep learning for tissue outcome prediction in acute stroke patients can outperform both threshold-based and the leading traditional machine learning method (random decision forests). Furthermore, spatiotemporal deep learning models using CT perfusion concentration-time curves perform as well as, and even insignificantly better, compared to using deconvolved residual curves or perfusion parameter maps, thereby eliminating the requirement for deconvolution. Overall, the best performance was achieved by the proposed CTC-Causal model, which used a causal temporal network for feature extraction in conjunction with the (non-deconvolved) CTP concentration-time curve data.

## Data availability statement

The raw data supporting the conclusions of this article will be made available by the authors, without undue reservation.

## Ethics statement

The studies involving human participants were reviewed and approved by Ethics committee of the Hamburg Chamber of Physicians, Hamburg, Germany and were conducted in compliance with the Declaration of Helsinki. Written informed consent for participation was not required for this study in accordance with the national legislation and the institutional requirements.

## Author contributions

AW: study conception and design, analysis and interpretation of data, drafting the manuscript, and revising it critically. MW and KA: study design and revising the manuscript critically. FF and JF: acquisition of data and revising the manuscript critically. NF: study conception and design, software, drafting the manuscript, and revising it critically. All authors contributed to the article and approved the submitted version.

## Footnotes

1
https://www.alglib.net

## Supplementary material

Supplementary Figure 1 Spearman’s Rho values for the correlation of each convolutionally-derived feature map with the four traditional perfusion parameters. RC, residual curve; CTC, concentration-time curve; CTP, computed tomography perfusion imaging; CBF, cerebral blood flow; CBV, cerebral blood volume; MTT, mean transit time; Tmax, time to maximum of the residual curve; Abbreviated model names correspond to deep learning from: deconvolved residual curves with convolutional (RC-Simple) or causal convolutional (RC-Causal) feature extraction, and deep learning from source concentration-time curves (CTC-Causal).

Supplementary Figure 2 Visualization of the convolutional features learned for the deep learning model trained from source concentration-time curves (CTC-Causal). Learned feature maps are normalized to the range [0, 1] for visualization. Traditional perfusion parameters are shown for comparison. CTP, computed tomography perfusion imaging; CBF, cerebral blood flow; CBV, cerebral blood volume; MTT, mean transit time; Tmax, time to maximum of the residual curve. Features 3 and 5 of the IV model contain no non-zero values for the patient shown. Other feature maps, which appear mostly black, typically have only a small handful of high-intensity voxels, which may not appear in the image slice shown.
