# Supplementary figures and images for: Predicting the tissue outcome of acute ischemic stroke from acute 4D computed tomography perfusion imaging using temporal features and deep learning
Source: Front Neurosci. 2022 Nov 4;16:1009654. doi: 10.3389/fnins.2022.1009654 (PMC9672821; doi:10.3389/fnins.2022.1009654)

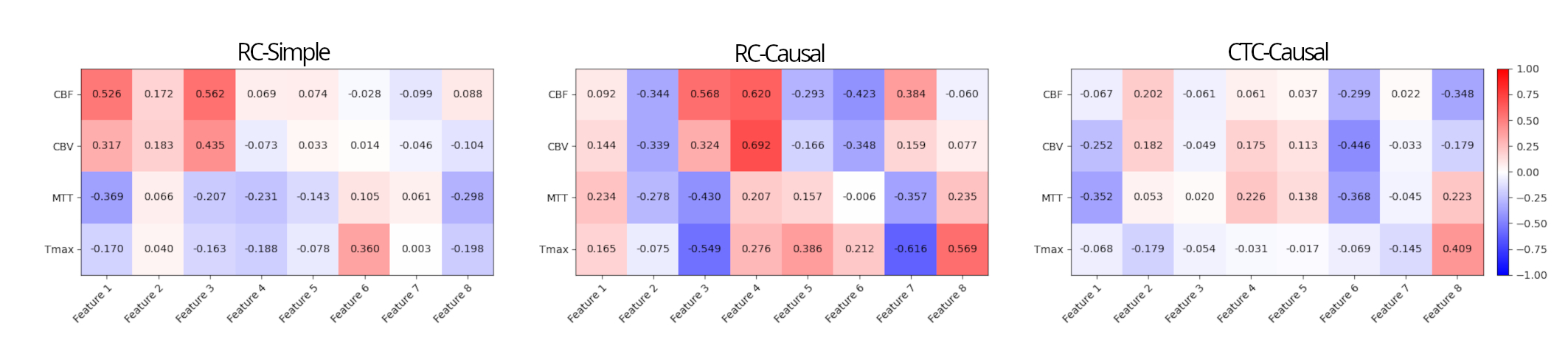

Supplement: Supplementary Figure 1 — Spearman’s Rho values for the correlation of each convolutionally-derived feature map with the four traditional perfusion parameters. RC, residual curve; CTC, concentration-time curve; CTP, computed tomography perfusion imaging; CBF, cerebral blood flow; CBV, cerebral blood volume; MTT, mean transit time; Tmax, time to maximum of the residual curve; Abbreviated model names correspond to deep learning from: deconvolved residual curves with convolutional (RC-Simple) or causal convolutional (RC-Causal) feature extraction, and deep learning from source concentration-time curves (CTC-Causal). [file Image_1.JPEG]

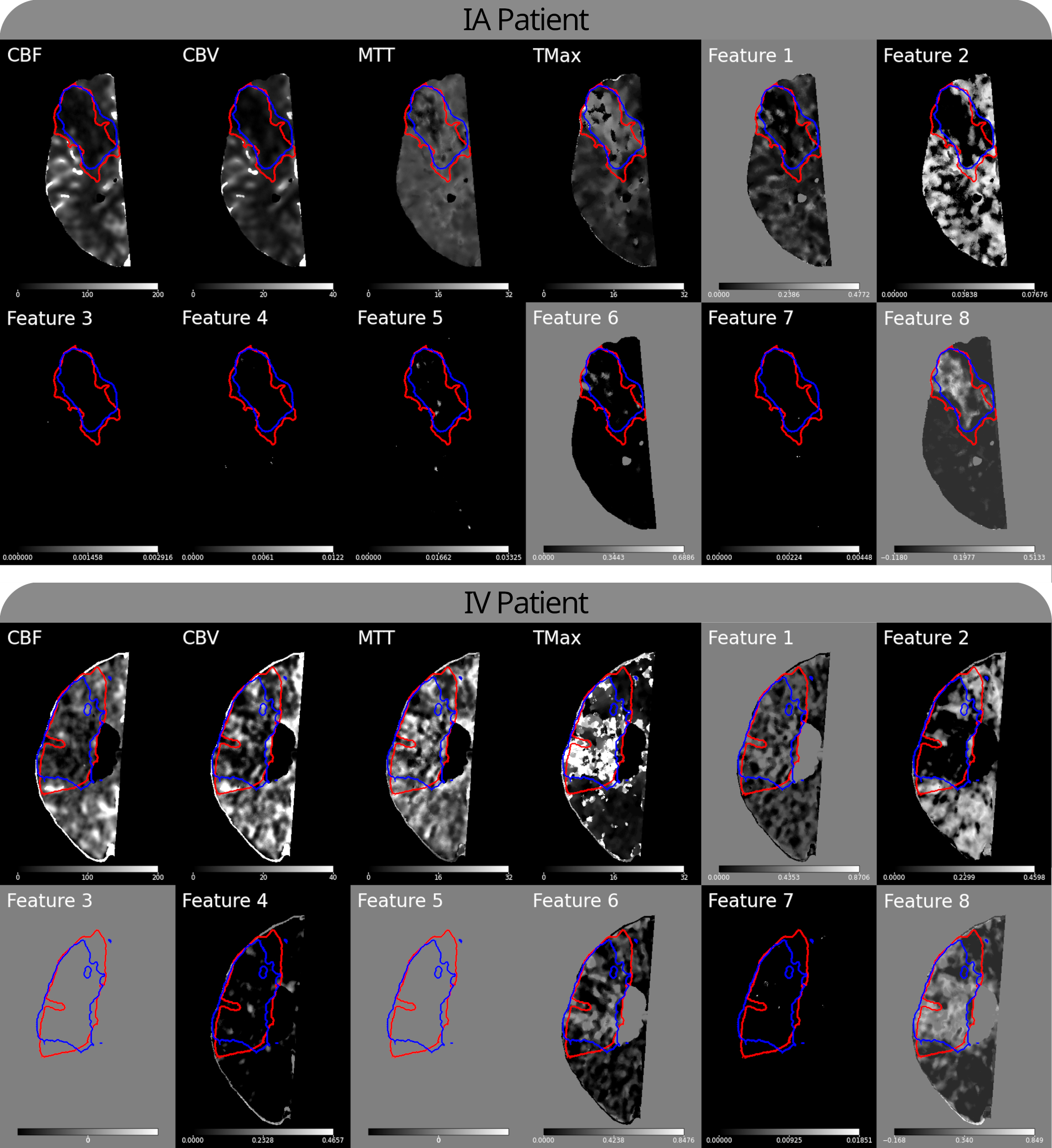

Supplement: Supplementary Figure 2 — Visualization of the convolutional features learned for the deep learning model trained from source concentration-time curves (CTC-Causal). Learned feature maps are normalized to the range [0, 1] for visualization. Traditional perfusion parameters are shown for comparison. CTP, computed tomography perfusion imaging; CBF, cerebral blood flow; CBV, cerebral blood volume; MTT, mean transit time; Tmax, time to maximum of the residual curve. Features 3 and 5 of the IV model contain no non-zero values for the patient shown. Other feature maps, which appear mostly black, typically have only a small handful of high-intensity voxels, which may not appear in the image slice shown. [file Image_2.JPEG]
